# Defects in early synaptic formation and neuronal function in Prader-Willi syndrome

**DOI:** 10.1038/s41598-023-39065-x

**Published:** 2023-07-25

**Authors:** Shuhei Soeda, Daiki Ito, Tomoe Ogushi, Yui Sano, Ryosuke Negoro, Takuya Fujita, Ryo Saito, Hideo Taniura

**Affiliations:** 1 Laboratory of Neurochemistry, College of Pharmaceutical Sciences, Ritsumeikan University, 1-1-1 Noji Higashi, Kusatsu, Shiga 525-8577 Japan; 2 Laboratory of Molecular Pharmacokinetics, College of Pharmaceutical Sciences, Ritsumeikan University, Kusatsu, Shiga 525-8577 Japan; 3 Mitsubishi Tanabe Pharma Corporation, Kamoshida, Aoba, Yokohama, 227-0033 Japan

**Keywords:** Biochemistry, Cell biology, Developmental biology, Genetics, Molecular biology, Neuroscience, Stem cells

## Abstract

Prader-Willi syndrome (PWS), which is a complex epigenetic disorder caused by the deficiency of paternally expressed genes in chromosome 15q11-q13, is associated with several psychiatric dimensions, including autism spectrum disorder. We have previously reported that iPS cells derived from PWS patients exhibited aberrant differentiation and transcriptomic dysregulation in differentiated neural stem cells (NSCs) and neurons. Here, we identified SLITRK1 as a downregulated gene in NSCs differentiated from PWS patient iPS cells by RNA sequencing analysis. Because SLITRK1 is involved in synaptogenesis, we focused on the synaptic formation and function of neurons differentiated from PWS patient iPS cells and NDN or MAGEL2 single gene defect mutant iPS cells. Although βIII tubulin expression levels in all the neurons were comparable to the level of differentiation in the control, pre- and postsynaptic markers were significantly lower in PWS and mutant neurons than in control neurons. PSD-95 puncta along βIII tubulin neurites were also decreased. Membrane potential responses were measured while exposed to high K^+^ stimulation. The neuronal excitabilities in PWS and mutant neurons showed significantly lower intensity than that of control neurons. These functional defects in PWS neurons may reflect phenotypes of neurodevelopmental disorders in PWS.

## Introduction

PWS is a multisystemic complex genetic disorder caused by dysfunction of the genes located on the chromosome 15q11-q13 imprinted region. The PWS locus contains several maternally silenced genes encoding MKRN3, MAGEL2, NECDIN, C15ORF2, and SNURF-SNRPN as well as noncoding genes^1^. MAGEL2, which is a gene with 51% amino sequence similarity to necdin, is expressed predominantly in the brain^2^. The NDN gene, encoding the MAGE family protein necdin, maps to the PWS chromosome region and is highly expressed in mature hypothalamic neurons^3^. The PWS phenotypes include poor sucking and feeding difficulties during early infancy (6–9 months) which impact overall health containing developmental delay or decreased growth velocity. The other features contain hyperphagia resulting in obesity from 2 to 4 years, and short stature caused by a growth hormone deficiency^4^. There are three main genetic subtypes in PWS^5^: paternal 15q11-q13 deletion (65–70% of cases), maternal uniparental disomy (UPD) 15 (20–25% of cases), and imprinting defect (1–3%). Our previous work demonstrated that iPS cells generated from PWS patients with abnormal methylation of 15q11-q13 (M-iPWS cells), in which both alleles are methylated, and with deletion of genes in 15q11-q13 (iPWS cells) exhibited both similar aberrant differentiation and transcriptomic dysregulation of NSCs and mature neurons^6,7^. Here, we identified Slit and Trk-like family member 1 (SLITRK1) as a differentially expressed gene between NSCs derived from control and PWS patient iPS cells by RNA sequencing analysis. Because SLITRK1 is involved in synaptogenesis, we focused on the synaptic formation and function of M-iPWS neurons and compared them to neurons from NDN or MAGEL2 single gene defect mutants generated by genome editing.

In adults, PWS patients have significant behavioral problems with obsessive–compulsive and psychosis characteristics^8^. In addition, PWS is associated with several psychiatric symptoms that can be connected to autism spectrum disorder (ASD). The pathophysiological and molecular mechanisms of PWS phenotypes have not been fully elucidated. Interestingly, it is well known that human chromosome 15q11-13 duplication leads to ASD^9^. Recently, ASD has been considered a disease of the synapse^10^. Given that PWS is caused by silencing of chromosome 15q11-13, synapse formation may be the key to understanding the mechanism of ASD phenotypes in PWS^11^. Most excitatory synapses are located on dendritic spines, which are small conformations that undergo dramatic changes during development. Post-synaptic density protein (PSD-95) is a member of the membrane-associated guanylate kinase (MAGUK) family, which is on the spine to maintain a balance between excitation and inhibition in the brain^10^. Although it is well known that the maturation of spines containing PSD-95 correlates with ASD phenotypes^12,13^, the molecular mechanism remains unclear. We focused on changes in spine density and impairment of membrane depolarization with immature synapse formation in neurons from NDN and MAGEL2 deletion mutants and M-iPWS neurons.

## Materials and methods

### Cell culture

Two sets of human iPS cells were provided by RIKEN BioResource Center (Tsukuba, Ibaraki, Japan). Normal human iPS cells from nasal epithelial cells (Nips cells; control iPS cells)^14^; iPS cells derived from the skin fibroblasts of a patient with PWS (iPWS cells) that had a paternal 15q11-q13 deletion and were created with the Sendai virus vector (DNAVEC Inc; Cyto Tune TMiPS vector) that individually carried OCT3/4, SOX2, KLF4, and c-MYC. Human iPS cells derived from a healthy individual (WT-iPS in Fig. 2A) and a patient with PWS (HPS2846: M-iPWS) with abnormal methylation were created with the pCE-hOCT3/4, pCE-hSK, pCE-hUL, pCE-mp53DD, and pCXB-EBNA1 vectors from peripheral blood mononuclear cells^15^. The iPS cells were maintained in feeder-free conditions and cultured in Cellartis DEF-CS medium (Takara Bio, Inc.) containing Cellartis DEF-CS 500 additives (DEF-CS GF-1 and GF-2) on-coated dishes with Cellartis DEF-CS COAT-1 (Takara Bio, Inc.). The iPS cells were passaged after reaching 70–80% confluence (every 3–5 days). To prevent cell death, iPS cells were cultured in medium containing the ROCK inhibitor Y27632 (Wako Pure Chemical Industries, Ltd) after cell passage.

### Genetic engineering of human induced pluripotent stem cells (hiPSCs)

hiPSCs (WT-iPS, clone #1210B2)^16^ were maintained in StemFit^®^ AK02N medium and incubated at 37 °C in 5% CO_2_. *NECDIN*- and *MAGEL2*-targeting crRNAs were designed using the CRISPR gRNA Design tool (https://www.atum.bio) and CHOPCHOP software (http://chopchop.cbu.uib.no). Alt-R^®^ CRISPR‒Cas9 crRNAs and Alt-R^®^ CRISPR‒Cas9 tracrRNA with ATTO™ 550 fluorescent dye (Integrated DNA Technologies (IDT), Coralville, IA) were fused together to form a single guide RNA (sgRNA) according to the manufacturer’s instructions. Thereafter, sgRNA and Alt-R^®^ S.p. HiFi Cas9 Nuclease 3NLS (IDT, Coralville, IA) was incubated with Cas9 PLUS™ Reagent (Thermo Fisher Scientific, Waltham, MA) to form a ribonucleoprotein (RNP) complex. The RNP complex was then transfected into dissociated hiPSCs using Lipofectamine™ CRISPRMAX™ Cas9 Transfection Reagent (Thermo Fisher Scientific, Waltham, MA), which was performed in a 1.5 mL microtube. The transfectants were subsequently cultured in StemFit^®^ AK02N containing 10 μM CultureSure^®^ Y-27632 (FUJIFILM Wako Pure Chemical Co., Osaka, Japan). To confirm the targeted mutations, the target region of sgRNA was amplified from the genomic DNA of whole cell populations using a specific primer set, which are also listed below, and the Alt-R^®^ Genome Editing Detection Kit (IDT, Coralville, IA). Thereafter, these whole cell populations were isolated by single-cell cloning, and the target region of sgRNA was confirmed by genomic DNA sequencing (FASMAC Co., Ltd., Atsugi, Japan). NDN sequence primers, forward primer (5'-) GCAGAGCCCTCCTCTAGGC, reverse primer (5'-) ACTTCTTGTAGCTGCCGATGAC, which reads 281 bp; MAGEL2 sequence primers, forward primer (5'-) CCACGTAGGCATTCTCTTCTCT, reverse primer (5'-) CAATGAAGCCTGCAAGTCAA, which reads 248 bp products.

### Induction of neural stem cells and neurons from iPS cells

iPS cells cultured in feeder-free conditions were split into 6-well plates coated with Geltrex matrix (Geltrex™ LDEV-Free Reduced Growth Factor Basement Membrane Matrix, Gibco) at a density of 2 × 10^5^ cells/well. One day after splitting, the culture medium was changed to PSC neural induction medium (neurobasal medium containing 2% neural induction supplement, Gibco). The medium was changed every 2 days from Day 0 to 7. On Day 7 of neural induction, primitive neural stem cells (NSCs) were dissociated with Accutase (StemPro Accutase cell dissociation reagent, Gibco) and replated on Geltrex matrix-coated 10.0 cm dishes with all NSCs in the wells in NSC expansion medium (49% Neurobasal medium, 49% Advanced DMEM/F12 and 2% neural induction supplement). The NSC expansion medium was changed every other day until the cells reached confluence at 6 days after plating. To maintain an undifferentiated state, induced NSCs were grown in complete StemPro NSC SFM medium, which contained Knockout DMEM/F-12, 2 mM GlutaMax-I (Gibco), 20 ng/mL recombinant human fibroblast growth factor-basic (FGFb), 20 ng/mL recombinant human epidermal growth factor (EGF), and 2% StemPro neural supplement (Gibco; Thermo Fisher Scientific). NSCs were then plated on Geltrex matrix-coated dishes with StemPro NSC SFM medium. When cells were 90% confluent, cells were dissociated using StemPro Accutase cell dissociation reagent (Gibco) and plated onto 10 µg/ml Laminin mixed Matrigel (Corning)-coated 6-well plates at 3 × 10^5^ cells/well for real-time RT‒PCR or 1 × 10^5^ cells/well for immunofluorescence. For differentiation into neurons, the NSC medium was changed to neuronal maturation medium based on the B-27 Plus Neuronal Culture System (Gibco) containing 2% B27 plus supplement, 2 mM GlutaMax-I, CultureOne Supplement (Gibco), which promotes synapse formation, and 0.2 mM ascorbic acid. The medium was changed every 3 days from Day 0 to Day 7 or Day 14.

### Realtime RT‒PCR analysis

Total RNA was isolated with IsogenII reagent (Nippon Gene Co., Ltd), and cDNA was synthesized from total RNA (1 µg) using a Transcriptor First Strand cDNA Synthesis Kit (Roche Life Science). PCR analyses were performed in PowerUp SYBR Green Master Mix (Applied Biosystems) containing 2 µL of cDNA and individual primers on a Step One Plus system (Applied Biosystems). All primers used for real-time PCR in this study are listed in Table 1^17–26^.

**Table 1:** Oligonucleotide sequences of realtime RT‒PCR primer sets.

Name of genes	Forward (5'-3')	Reverse (5'-3')	References
NECDIN (NDN)	GAGAGCGCCGTCTGGAAC	ACTGCTGCGAGGGTAGTGG	Ref.^17^
MAGEL2	GGTCCAAAGCCCTATCCAG	CACTTGCGACCTCAGACAC	Ref.^17^
NPTX1	TTGTCCTCATGCACACGAAGCAGC	ACACGCACACACAGATCCTCTCAC	Ref.^18^
NLGN3	ACATTGCTGGGCACCTGTAG	GCACCCCTTAGCTTCCCAAA	
NLGN4X	GAAGCCCGTCATGGTCTATATC	AGTATTCCCAGACGGTAGTTAATG	Ref.^19^
SLITRK1	AACGTTACAGGGGACGTTTG	CCCAGAAAAGTCTGCTTTCG	Ref.^20^
SOX4	CAGAAGGGAGGGGGAAACATA	GAATCGGCACTAAGGAGTTGGT	Ref.^21^
SEMA3B	TTCTTTCGTGAGACGGCGGTA	CCCTGGAAGATGCTGCTGGA	Ref.^22^
βIII-Tubulin	GCGAGATGTACGAAGACGAC	TTTAGACACTGCTGGCTTCG	Ref.^23^
MAP2	AATCAGCTCTGGCTCCCAGT	AGTGGGTGTTGAGGTACCAC	Ref.^24^
SYN1	TGAAGCCGGATTTTGTGCTGA	GACCAAACTGCGGTAGTCTCC	Ref.^25^
PSD-95	TCACAACCTCTTATTCCCAGCA	CATGGCTGTGGGGTAGTCG	Ref.^25^
SCN2B	ATAGGAATGGCTTGGTGCAGT	AGTGCATCAGCAAGCTTCAAT	Ref.^26^
SCN3B	AATAGTGCTTGGTGTGCTTGC	ACTGCATGGCACACTAAAGGT	Ref.^26^
SCN4B	AGGCATCTGGAGACTGAGAAA	ATGGCAGCTGTGTGTGACACT	Ref.^26^
GAPDH	TGCCTCCTGCACCACCAACT	CGCCTGCTTCACCACCTTC	Ref.^7^

### Western blotting

A total of 1 × 10^6^ NSCs were transferred to Matrigel-laminin-coated 6 cm culture dishes (Nunclon). After 11 days of neuronal induction, whole cell lysates were obtained by lysing cell pellets in CSK buffer (10 mM PIPES pH 6.8, 100 mM NaCl, 300 mM sucrose, 3 mM MgCl_2_, 1 mM EGTA, 0.5% Triton X-100). The cell lysates were centrifuged at 10,000 × *g* for 5 min at 15 °C, and the supernatants were used as lysates. The protein concentration was determined using the Bradford assay. The lysates were then mixed with SDS sample buffer and boiled for 5 min at 95 °C in preparation for SDS‒PAGE.

### Next-generation RNA sequencing (RNA-seq) analysis

Total RNA was isolated using NucleoSpin RNA Plus XS (MACHEREY–NAGEL) from Nips, iPWS, M-iPWS, Nips NSC, iPWS NSC, and M-iPWS NSC cells according to the manufacturer’s instructions. RNA sequencing was ordered from Macrogen (Japan) and performed with a NovaSeq 6000 System. In this study, Homo sapiens whole transcriptome sequencing was performed to examine the differentially expressed gene (DEG) profiles and to perform gene annotation on a set of useful genes based on gene ontology (GO) pathway information. The DEG analysis was performed on 4 comparison pairs, Nips to iPWS or M-iPWS cells and Nips NSCs to iPWS NSCs or M-iPWS NSCs, as requested using edgeR. The results showed 2607 genes that satisfied |fc|> = 2 and exactTest raw p value < 0.05 conditions in at least one of the comparison pairs.

### Immunofluorescence

NSCs were induced to differentiate into neuronal cells for 14 days on 4-well chamber slides (Thermo Scientific Nunc) coated with Matrigel in laminin solution. Cells were fixed with 4% paraformaldehyde (Wako Pure Chemical Industries) for 20 min at room temperature. After washing with phosphate-buffered saline (PBS), the cells were permeabilized by adding 0.1% Triton X-100 and incubated for 15 min. Primary antibodies were diluted to 1 µg/mL for anti-βIII-tubulin (Promega, G7121) and anti-PSD-95 (0.5 µg/mL, Invitrogen, 51–6900) in PBS containing 5% donkey serum and incubated for 1.5 h. After washing, the cells were incubated with secondary antibodies: Alexa 488 anti-mouse IgG (2 µg/mL, Thermo Fisher Scientific) and Alexa 555 anti-rabbit IgG (2 µg/mL, Thermo Fisher Scientific) for 1.0 h. Cell nuclei were stained with DAPI (300 nM). Photographs were obtained using an EVOS FL fluorescence microscope (Thermo Fisher). PSD-95 localization on neurons was imaged on a BZ-X700 (KEYENCE) using Z-stacking to image the steric neuron via BZ-X700 Analyzer software (KEYENCE JAPAN). The images were acquired with a 100 × magnification (Nikon; NA, 1.45) fluorescence objective lens with 8–15 z steps per stack at a step interval of 0.4 µm.

### Measurement of membrane depolarization

NSCs were transferred to poly-L-ornithine- and laminin-coated 96-well plates. The NSCs were induced to differentiate into neuronal cells for 7 days with the B27 Plus Neuronal Culture System (Thermo Fisher Scientific). After 7 days of induction, neuronal cells were loaded with a FluoVolt Membrane Potential Kit (Thermo Fisher Scientific)^27^, which is a fluorescence resonance energy transfer (FRET)-based voltage sensor. The neuronal cells were incubated with the dye, which was dissolved in Live Cell Imaging Solution (LCIS, Life Technologies) containing 20 mM glucose for 30 min. After two washes, the cells were incubated in LCIS containing Neuro Background Suppressor to block the background. Neural cells were stimulated by adding an equal volume of isotonic potassium chloride (KCl) solution: 140 mM KCl, 5 mM NaCl, 1.8 mM CaCl_2_, 1.0 mM MgCl_2_, 20 mM HEPES, and 20 mM glucose, pH 7.4. The measurement of the fluorescence was performed with a microplate reader (SH-9500 Lab, CORONA ELECTRIC Co., Ltd. Japan) every 30 s. The measurement was maintained at 37 °C with an excitation wavelength of 490 nm and an emission wavelength of 535 nm. The dotted line data display the average of 25 measurement points in a well with neurons (Fig. 5A). KCl was added 120 s after the start of the experiment using the automated drug delivery system, and fluorescence recording was continued for an additional 360 s. Membrane fluorescence was expressed as the difference in activity between the membrane fluorescence of neurons and baseline levels. The baseline level was the activity of buffer without cells in wells after washing with FluoVolt dye.

### Experimental design and statistical analysis

Statistical analyses were conducted using GraphPad Prism 9. Comparisons of two groups were performed by one-way analysis of variance (ANOVA) with multiple comparisons test (Tukey’s or Kruskal‒Wallis test). The statistical test and number of independent experiments used for each analysis are indicated in each figure legend.

## Results

### Next-generation RNA sequencing analysis between NSCs derived from control iPS cells and iPWS or M-iPWS cells

**Figure 1:**
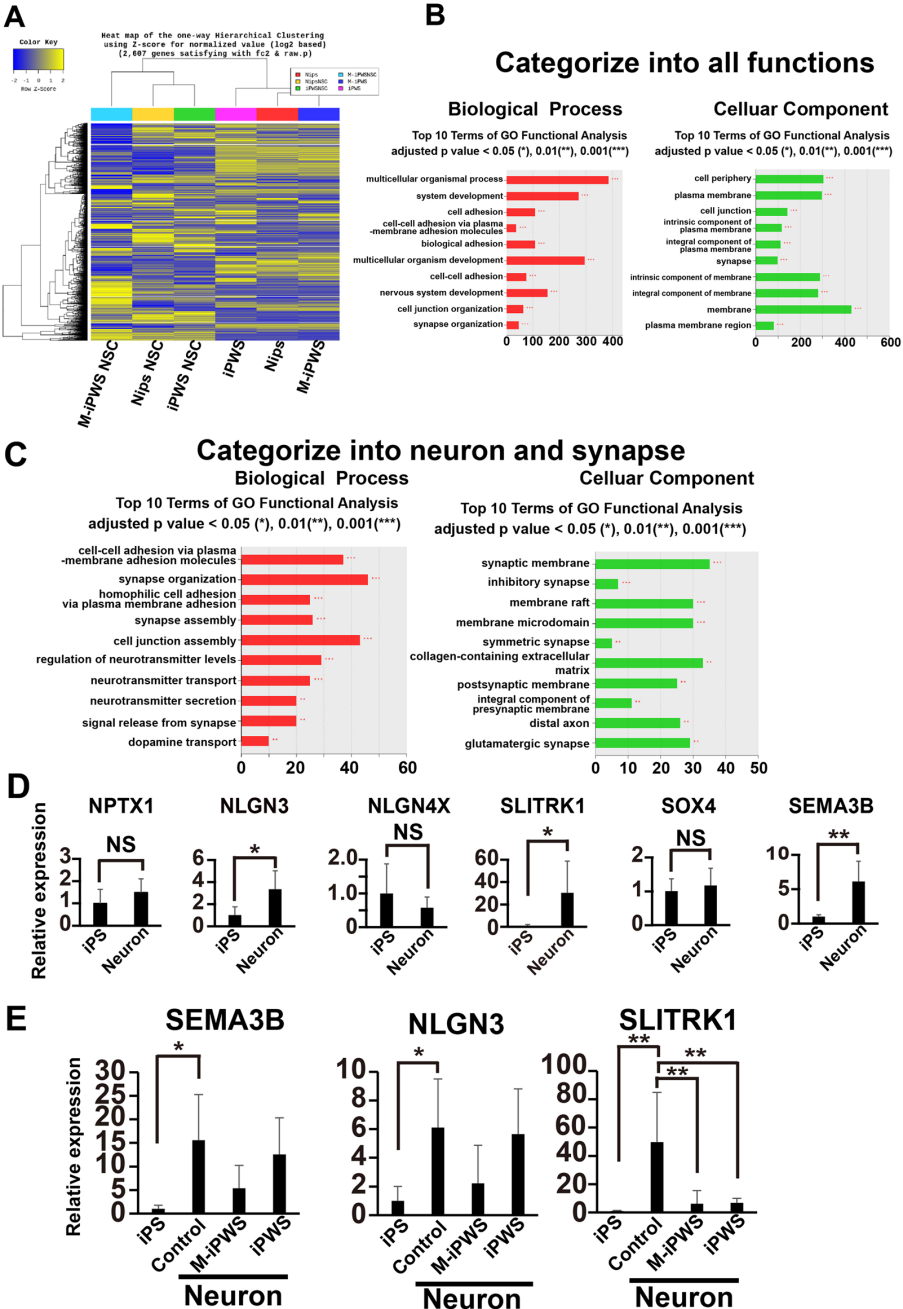
RNAseq analysis reveals enrichment in differentially expressed downregulated transcripts in M-iPWS NSCs. (**A**) Heatmap of transcripts identified by DEG analysis showing expressed genes between control (Nips) iPS cells, M-iPWS cells, and iPWS cells. These data displayed iPS cells and NSC differentiated cells. (**B,C**) List of differentially downregulated genes expressed in M-iPWS NSCs versus control NSCs with the category of all function in 1B and neuron and synapse in 1C. (**D**) Quantitative mRNA analysis of neural development- and synaptogenesis-related genes in neurons (14 days of differentiation), including NPTX1, NLGN3, NLGN4X, SLITRK1, SOX4, and SEMA3B, selected by the Metascape tool. Each value was normalized to the GAPDH value and expressed as the fold induction from the levels in undifferentiated control iPS cells. Values represent the mean ± S.D. All the data were evaluated by unpaired student’s t test, *p < 0.05, **p < 0.01(all data, n = 5). NS showed a no significant difference. (**E**) Quantitative mRNA analysis of NLGN3, SLITRK1, and SEMA3B in control neurons, M-iPWS and iPWS neurons, which were 14 days of differentiation. Each value was normalized to the GAPDH value and expressed as the fold induction from the levels in undifferentiated control iPS cells. Values represent the mean ± standard deviation (S.D.) All the data were evaluated by one-way ANOVA followed by Tukey’s multiple comparisons test (SEMA3B and SLITRK1) or the Kruskal‒Wallis test followed by Dunn’s multiple comparisons test (NLGN3), *p < 0.05, **p < 0.01 (all data, n = 5).

To identify molecular changes specific to PWS, we investigated the significantly different transcripts in the control iPS cells versus iPWS or M-iPWS cells and NSCs derived from control iPS cells versus iPWS or M-iPWS cells. Here, we focused on analyzing the differential expression between NSCs derived from control iPS cells and iPWS or M-iPWS cells. These transcripts were evaluated with enrichment analysis based on the GO database with a significant gene list using the g: Plofiler tool (https://biit.cs.ut.ee/gprofiler/orth). The heatmap obtained from our data illustrates that the expression pattern of genes in M-iPWS NSCs was unique compared to that in control and iPWS NSCs (Fig. 1A). The DEG analysis showed that downregulated genes in M-iPWS NSCs were involved in multicellular organism processes, cell adhesion and junctions, synapse organization, and nervous system development (Fig. 1B,C). Among them, 98 genes were associated with nervous system functions. Furthermore, we selected 6 genes related to neural development and synaptogenesis with the Metascape tool^28^. The candidate genes were NPTX1, NLGN3, NLGN4X, SLITRK1, SOX4, and SEMA3B, which were selected with the function and ratio of expression levels to control NSCs. We performed mRNA expression analysis of these genes in control neurons compared to undifferentiated iPS cells (Fig. 1D). The results showed that NLGN3, SLITRK1, and SEMA3B were induced after neuronal differentiation more than three times compared to that of the control. Next, we checked the differential mRNA expression of these three genes among control, M-iPWS, and iPWS neurons (Fig. 1E). SEMA3B and NLGN3 showed the same expression levels in iPWS neurons as control neurons. SLITRK1 expression was downregulated in M-iPWS and iPWS neurons (Fig. 1E). SLITRK1 is involved in synaptogenesis, and many functional genes of synapses were decreased in the DEG data. These results suggested that SLITRK1 is a candidate molecule involved in neural dysfunction in PWS.

### Generation of NDN KO and MAGEL2 KO iPS cells

**Figure 2:**
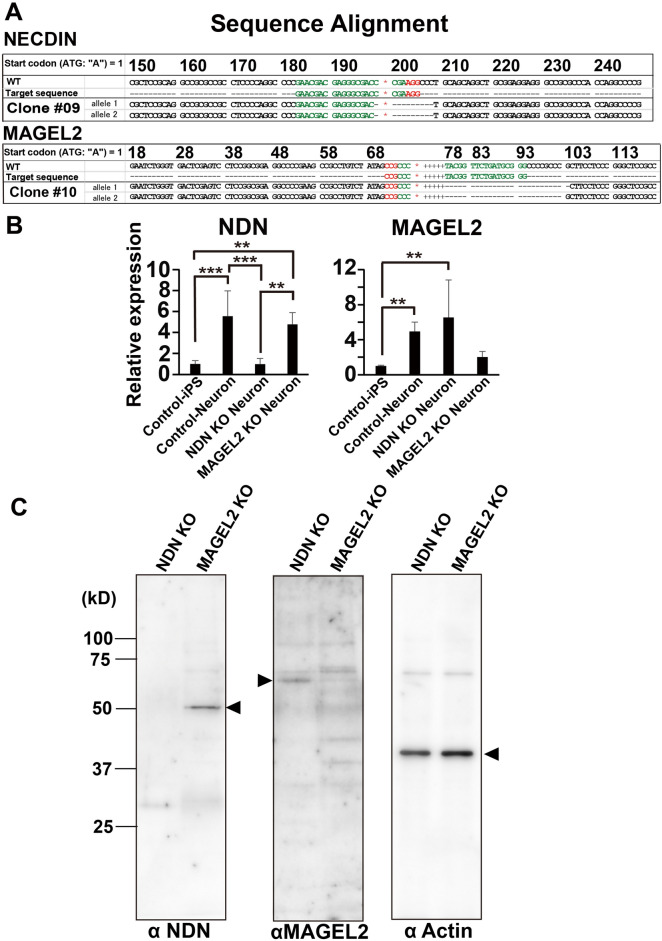
Generation of NDN KO and MAGEL2 KO iPS cells. (**A**) Genome sequencing was performed and aligned with the reference sequence (WT). NDN KO clone#9 showed a 10 bp deletion in both alleles. MAGEL2 KO clone #10 showed 26 and 25 bp deletions in each allele, respectively. Green parts indicated target sequence of sgRNA including PAM site (Red part). *PAM* protospacer adjacent motif. (**B**) Quantitative mRNA analysis of NDN and MAGEL2 in NDN and MAGEL2 KO neurons at 7 days of neural differentiation. Each value was normalized to the GAPDH value and expressed as the fold induction from the levels in undifferentiated control iPS cells. Values represent the mean ± standard deviation (S.D.) All the data were evaluated by one-way ANOVA followed by Tukey’s multiple comparisons test (NDN) or the Kruskal‒Wallis test followed by Dunn’s multiple comparisons test (MAGEL2), **p < 0.01, ***p < 0.001 (all data, n = 5). (**C**) Western blot analysis showing the protein expression levels of NDN and MAGEL2 in NDN and MAGEL2 KO neurons at 11 days of neural differentiation. Arrowheads showed specific bands of each antibody.

We generated NDN and MAGLE2 KO iPS cells from a normal individual iPS cell using gene editing technology. After NDN and MAGEL2 genome editing, 12 independent clones each were isolated, and genomic sequencing was performed and aligned with reference genome sequences (NDN: NC_000015.10 and MAGEL2: NC_000015.10). Two NDN KO clones have the same 10 bp deletion in both alleles, causing a frameshift and forming a premature stop codon (Fig. 2A). One MAGEL2 KO clone has 26 and 25 bp deletions in each allele, causing a frameshift and forming a premature stop codon in both alleles (Fig. 2A)^29^. To evaluate the KO clones, we analyzed the mRNA and protein expression levels of NDN and MAGEL2 (Fig. 2B,C). Both NDN and MAGEL2 mRNA were induced after neural differentiation and were decreased in neurons derived from NDN or MAGEL2 KO iPS cells, respectively. MAGEL2 protein expression was detected in neurons from NDN KO iPS cells but not in neurons from MAGEL2 KO iPS cells. In contrast, NDN protein expression was detected in neurons from MAGEL2 KO iPS cells but not in neurons from NDN KO iPS cells. These results confirmed that we generated NDN or MAGEL2 KO iPS clones with defective expression of NDN or MAGEL2, respectively.

### Neuronal differentiation and early stage of synaptic formation

**Figure 3:**
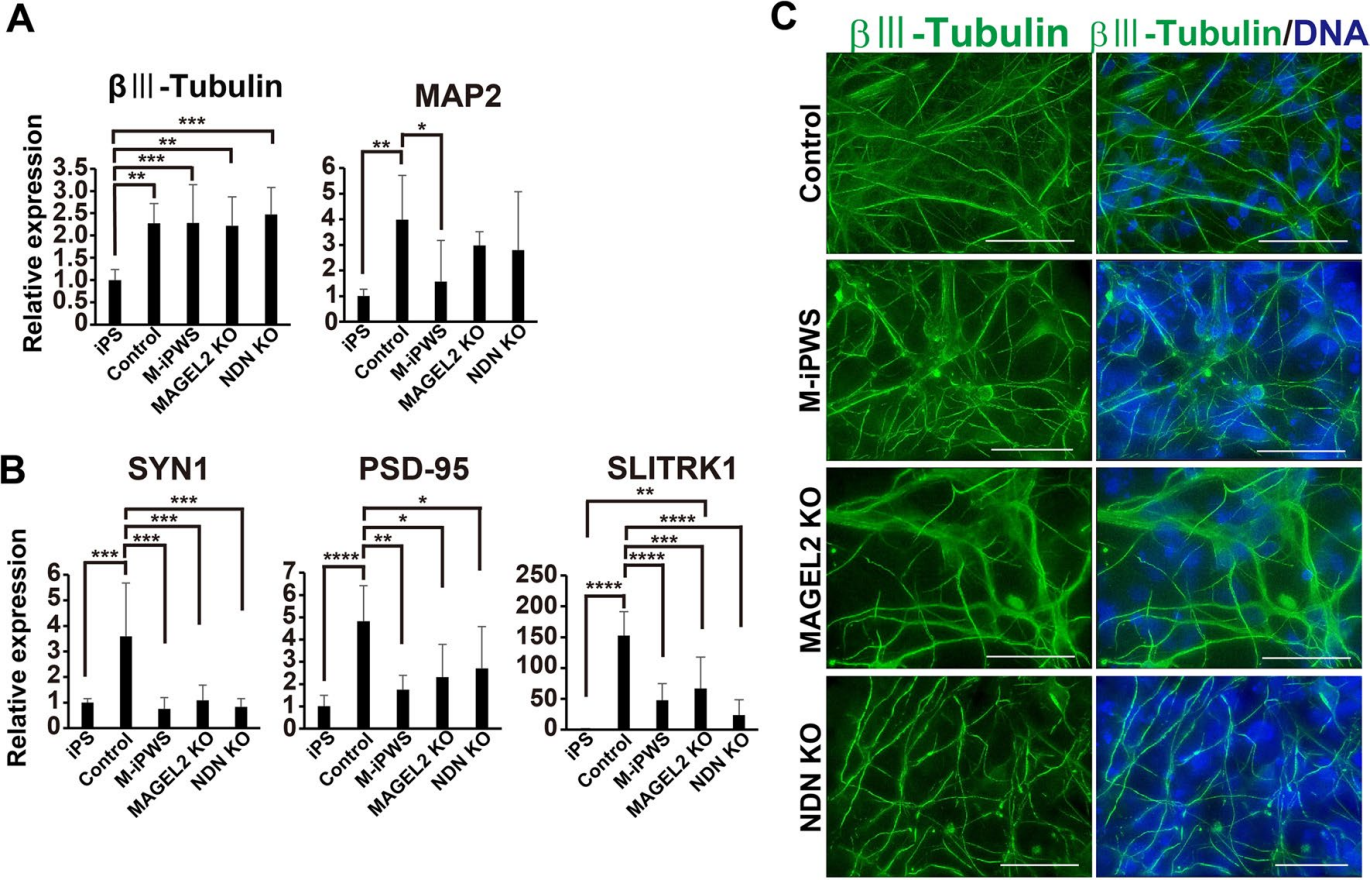
Neuronal differentiation induced from control, M-iPWS, NDN KO, and MAGEL2 KO iPS cells. Quantitative mRNA analysis of neuronal markers (ßIII-tubulin and MAP2) (**A**) and synapse-related genes (presynapse, SYN1; postsynapse, PSD-95 and SLITRK1) (**B**). Each value was normalized to the GAPDH value and expressed as the fold induction from the levels in undifferentiated control iPS cells. Values represent the mean ± S.D. All data were evaluated by one-way ANOVA followed by Tukey’s multiple comparisons test, *p < 0.05, **p < 0.01, ***p < 0.001, and ****p < 0.0001 (all data, n = 7). (**C**) 14-day differentiated neurons were immunostained with neuron (ßIII-tubulin) and DNA (DAPI). The scale bars represent 10 µm.

We examined neuronal differentiation from NDN KO and MAGEL2 KO iPS cells and compared to control iPS and M-iPWS cells. Another PWS-derived iPS cell, iPWS cells, a deletion type, has shown more severe impairment of differentiation to reduce βIII tubulin-positive neuron production. Therefore, we used M-iPWS cells, an abnormal methylation type, as PWS patient-derived iPS cells. Human iPS cells were differentiated into NSCs via a previously described method^6^. After 14 days of differentiation from NSCs, neurons derived from all 4 different iPS cells expressed βIII-tubulin at almost the same levels (Fig. 3A, Left). When immunofluorescent staining for βIII-tubulin was performed, all the neuron-derived control, M-iPWS, NDN KO and MAGEL2 KO cells were labeled in a similar fashion (Fig. 3C, Right). MAP2 expression was also highly increased in control neurons and in MAGEL2 KO and NDN KO neurons. Although βIII-tubulin expression levels in all differentiated neurons were comparably induced, M-iPWS neuron showed relatively low expression of MAP2, which was described previously (Fig. 3A, Left)^6^. Because SLITRK1 is involved in synaptogenesis, next, to evaluate synapse formation in neurons derived from iPS cells, we analyzed the mRNA expression of SYN1, a presynaptic marker, PSD-95, a postsynaptic marker, and SLITRK1 (Fig. 3B). Although the expression of these synaptic markers in control neurons was highly increased after neural differentiation, the expression in other neurons, such as M-iPWS, NDN KO and MAGEL2 KO neurons, was significantly lower than that in control neurons. Although SLITRK1 was identified by differential expression screening between control NSCs and NSCs from PWS patients, its expression was also downregulated in neurons from single gene KO (NDN KO and MAGEL2 KO) mutants.

### PSD-95 localization on neurons

**Figure 4:**
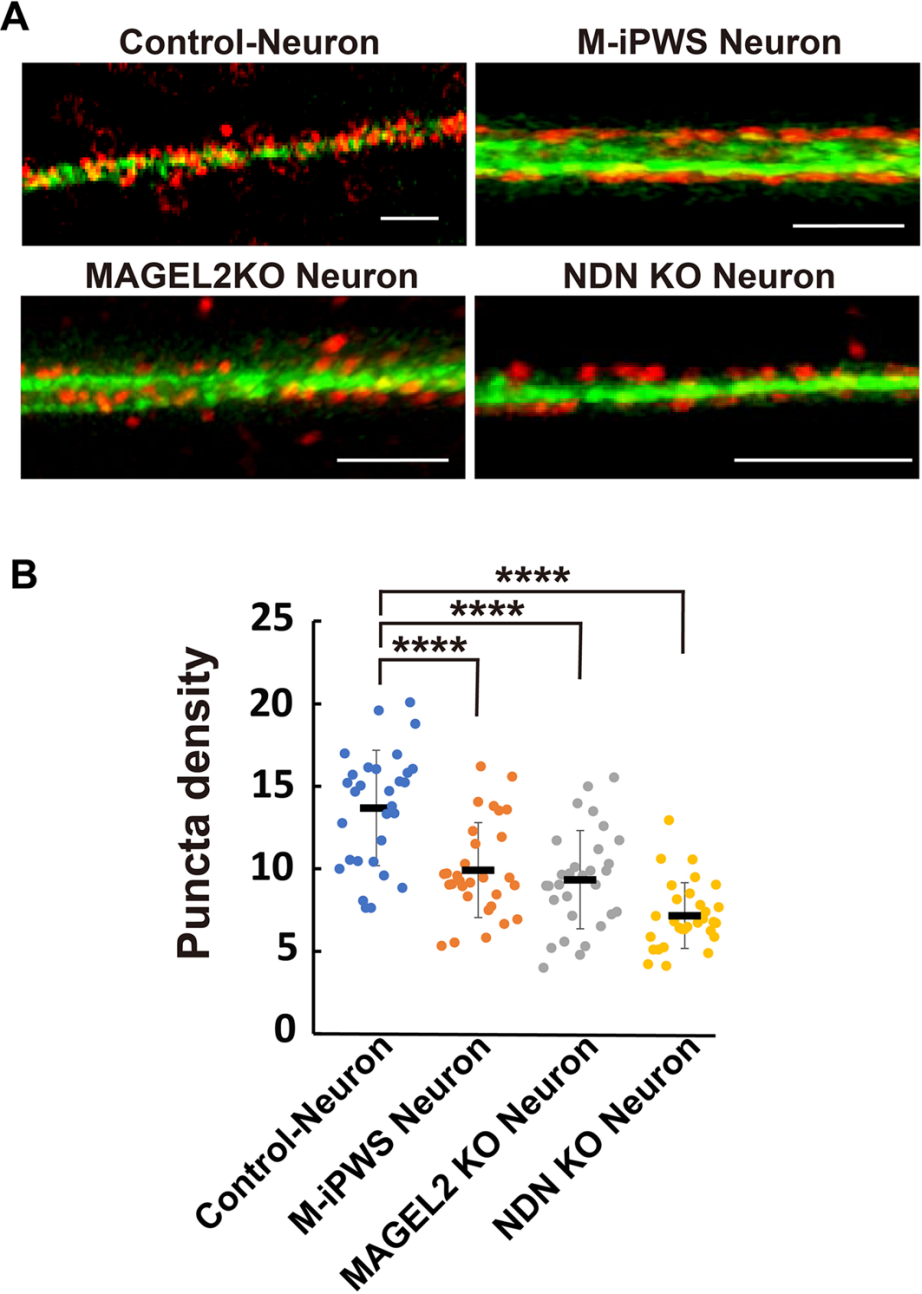
Synaptic density on neurons derived from control, M-iPWS, NDN KO, and MAGEL2 KO iPS cells. (**A**) Representative 2D reconstruction of PSD-95 puncta on βIII tubulin-positive dendrites for each neuron. Neurons are indicated in green, and colocalization of PSD-95 is indicated in red. All scale bars represent 5 µm. (**B**) Quantification of PSD-95 puncta on neurons. PSD-95 per 10 µm neuron: control, 14 ± 3.5; M-iPWS, 9.9 ± 2.9; MAGEL2 KO; 9.3 ± 3.0; NDN KO; 7.2 ± 2.0. Values represent the mean ± S.D. All data were evaluated by one-way ANOVA followed by Tukey’s multiple comparisons test, ****p < 0.0001 (all data, n = 30; independent neurons of several experiments).

Because synaptic marker expression was reduced in neurons derived from M-iPWS, NDN KO and MAGEL2 KO iPS cells, we examined the expression of the postsynaptic marker PSD-95 by immunofluorescence staining (Fig. 4A). βIII-tubulin-positive dendrites in neurons derived from M-iPWS, NDN KO and MAGEL2 KO iPS cells exhibited a reduced density of PSD-95 puncta. We measured PSD-95 puncta numbers at the tips of βIII-tubulin-positive dendrites. This is because the tip of dendrites is a high activity site that was a highly concentrated region of PSD-95. The density of PSD-95 puncta in M-iPWS, NDN, and MAGEL2 KO neurons was significantly lower than that in control neurons (control neurons vs. NDN KO, p < 0.0001; MAGEL2 KO vs. NDN KO, p < 0.05, Fig. 4B). Synaptic formation assessed by PSD-95 puncta on NDN KO neurons was most severely impaired among them (Fig. 4A,B). These results demonstrate that NDN or MAGEL2 single gene deletion could induce impairment of synaptogenesis in a similar fashion as PWS.

### The membrane potential in depolarized neuronal cells

**Figure 5:**
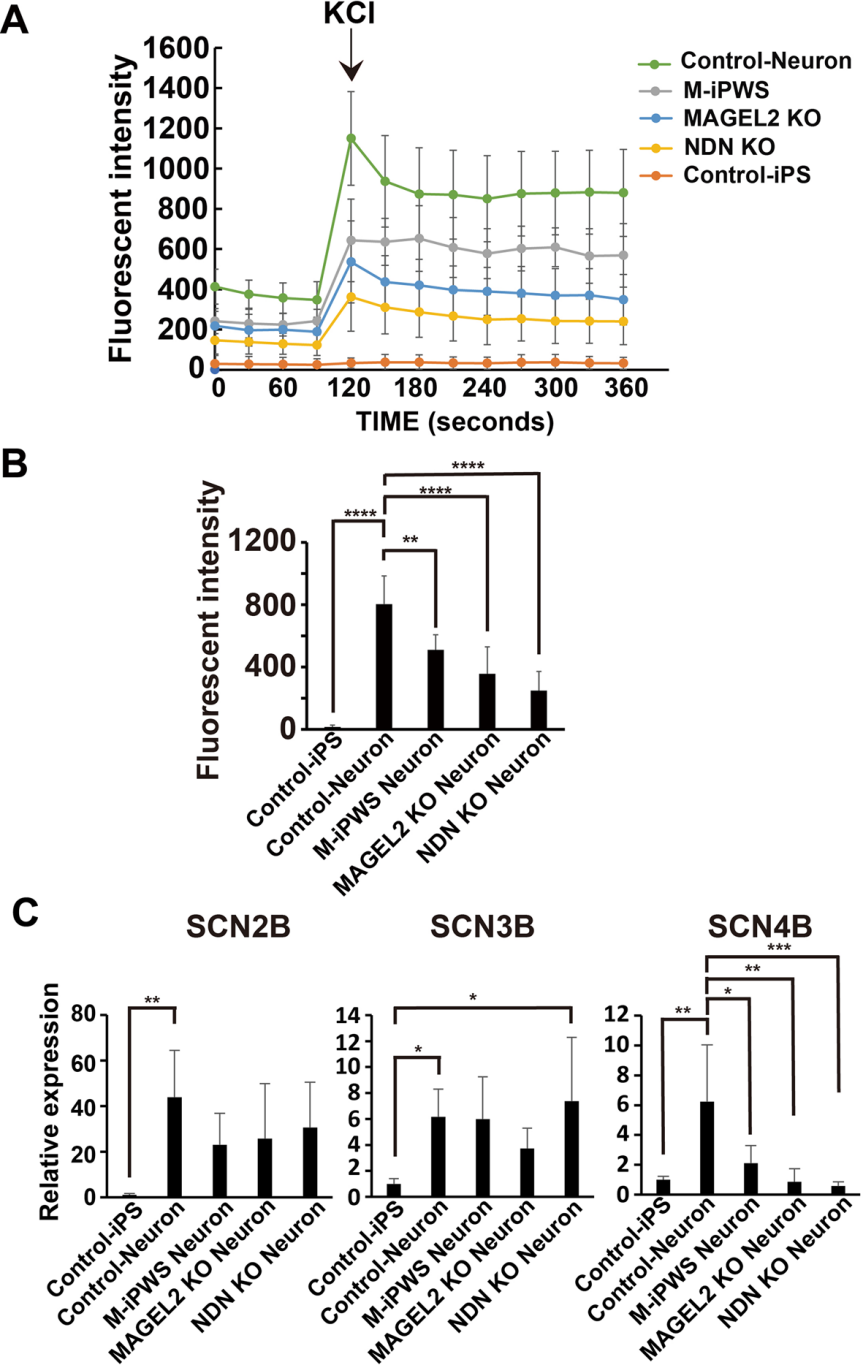
Effects of high K^+^ solution on the membrane potential of neurons from control, M-iPWS, NDN KO, and MAGEL2 KO iPS cells using Fluo Volt. (**A**) Kinetic analysis of neuronal membrane activity was detected in 96-well plates. After 120 s (black arrow) from monitoring onset, high K^+^ solution was added to depolarize the neuronal cells. Fluorescence intensity was measured at 535 nm/490 nm. Values represent the mean ± S.D. (**B**) The increased maximum responses of excitatory neurons from the baselines. Values represent the mean ± S.D. These data were evaluated by one-way ANOVA followed by Tukey’s multiple comparisons test, **p < 0.01, ****p < 0.0001 (n = 8). (**C**) Quantitative mRNA analysis of sodium channel subunits in neuronal cells. Each value was normalized to the GAPDH value and expressed as the fold induction from the levels in undifferentiated control iPS cells. Values represent the mean ± S.D. All data were evaluated by one-way ANOVA followed by Tukey’s multiple comparisons test (SCN2 and 4B) or the Kruskal‒Wallis test followed by Dunn’s multiple comparisons test (SCN3B), *p < 0.05, **p < 0.01, and ***p < 0.001 (all data, n = 5). All data for neurons in Fig. 5 were obtained after 7 days of differentiation from NSCs.

Next, we determined the electrophysiological maturation of iPS cell-derived neurons. Because membrane depolarization is evoked at synaptic sites, the excitability of neurons may be altered in neurons derived from PWS patients. Therefore, we analyzed the membrane potential in depolarized neuronal cells^30^. Membrane potential responses were measured using FluoVolt under the presence of high K^+^ solution. High K^+^ stimulation induced a rapid increase in fluorescence intensity in control neurons, indicating membrane depolarization, and the effect was sustained in the presence of high K^+^ solution, whereas no such response was observed in undifferentiated control iPS cells (Fig. 5A). However, the neuronal excitabilities in the other neurons from M-iPWS, NDN KO and MAGEL2 KO cells showed significantly lower intensity than that of control neurons, especially lowest in NDN KO neurons among them (Fig. 5A and control neurons vs. NDN KO, p < 0.0001; M-iPWS vs. NDN KO, p < 0.01 in Fig. 5B). The results of membrane potential recording are consistent with the decreased PSD-95 puncta observed in βIII tubulin-positive dendrites from neurons derived from M-iPWS, NDN KO and MAGEL2 KO iPS cells. Finally, we evaluated the expression levels of sodium channel transcripts SCN2B, 3B, and 4B related to depolarization in neurons. All 3 sodium channel transcripts were induced after neural differentiation (Fig. 5C). Among them, a significant decrease in SCN4B expression was observed in neurons from M-iPWS, NDN KON and MAGEL2 KO iPS cells compared with control neurons. NDN and MAGEL2, whose expression is deleted in PWS, contribute to the depolarization defect.

## Discussion

PWS is a genetic disorder characterized by the loss of paternal expression for several genes at the chromosome 15q11-q13 locus. A correlation between the disease phenotypes and underlying gene responsible within the PWS locus remains unclear. Here, we show that the early stage of synapse and spine formation is affected by silencing genes on 15q11-13 and two of the silencing genes, NDN and MAGEL2 gene deletions, resulting in suppressed depolarization. Interestingly, previous work on a 15q11-13 duplication mouse model also showed impaired spine formation^12,31^. The genes in the 15q11-13 region could be related to synapse and neuronal systems. Further investigation of the correlation between genes in 15q11-13 and disease phenotypes of synapses is needed. Remarkably, Schaaf-Yang syndrome (SYS) is also characterized by nonsense mutations in the MAGEL2 gene. The syndrome displays overlapping phenotypes with PWS, including ASD^32,33^. Our data showing the synaptic impairments in MAGEL2 deletion neurons may reflect the overlapping phenotypes with PWS and SYS.

Expression profiling in this study showed that SLITRK1 may affect early synapse formation in PWS neurons (Fig. 1A–E). We focused on SLITRK1, which is a transmembrane protein that is enriched in postsynaptic fractions, is localized to excitatory synapses and regulates synapse formation^34,35^. The overexpression of SLITRK1 in hippocampal neurons promotes the formation of excitatory and inhibitory synapses^34^. SLITRK1 is associated with Tourette’s syndrome, which displays obsessive–compulsive disorder^36^, and SlITRK1 KO mice show behavioral abnormalities^37^. Our data on the downregulated expression of SLITRK1 in NDN KO and MAGEL2 KO neurons suggested that SLITRK1 could be a key regulator of synapse formation related to the ASD phenotype in PWS (Fig. 3B). In fact, PWS and single gene deletion mutant neurons exhibited a reduced density of PSD-95 puncta and reduced expression of SYN1 and PSD-95 transcripts. The decreased PSD-95 puncta observed are consistent with the results of membrane potential recording. Our data indicate a potential neuronal network impairment associated with PWS pathology. We also demonstrated that SCN4B, but not SCN2B nor SCN3B, sodium channel transcripts, expression was significantly decreased in PWS and mutant neurons. The 9 voltage-gated sodium channel α subunits encoded by the SCN (1–10) A genes and 5 β subunits encoded by the SCN (1–4) B genes are known in mammals^38^. The SCN4B mRNA and protein are highly expressed in several neuronal populations^39^, especially the striatum and cerebellar Purkinje neurons, but are also highly expressed in other restricted brain regions^40^. SCNB2 and B4 form disulfide bonds with α subunits and resemble each other closely^41^. Interestingly, α subunits in the absence of β2 or β4 fail to be enriched in synaptosomes^42^. These findings suggested that SCN4B expression in neurons may affect the localization of SCN2B and α subunits, resulting in synapse impairment of depolarization. Finally, our data showed that all synaptic phenotypes were most severely affected by NDN deletion (Figs. 3B, 4B, and 5B). Surprisingly, PWS neurons showed milder phenotypes than NDN and MAGEL2^43,44^ single gene KO neurons. Other silencing genes in PWS may affect the expression and/or activation of downstream factors, such as SLITRK1. In summary, we found that synaptic defects containing PSD-95 resulted in reduced depolarization with NDN and MAGEL2 gene deletion and gene silencing in PWS (Supplementary Fig. S1).

## Supplementary Information

Supplementary Figure S1.

## Data Availability

All data generated and analyzed during this study are either included in the published article itself (or available from the corresponding author upon reasonable request).
