# Supplementary material for: Defects in early synaptic formation and neuronal function in Prader-Willi syndrome
Source: Sci Rep. 2023 Jul 25;13:12053. doi: 10.1038/s41598-023-39065-x (PMC10368700; doi:10.1038/s41598-023-39065-x)
Supplement: Supplementary file 1 — Supplementary Figure S1. [file 41598_2023_39065_MOESM1_ESM.pdf]

Defects in early synaptic formation and neuronal function in Prader-Willi syndrome

Shuhei Soeda[1], Daiki Ito[1], Tomoe Ogushi[1], Yui Sano[1], Ryosuke Negoro[2], Takuya Fujita[2], Ryo Saito[3], Hideo Taniura[1]

[1]Laboratory of Neurochemistry, College of Pharmaceutical Sciences, Ritsumeikan University, Shiga, 525-8577, Japan

[2]Laboratory of Molecular Pharmacokinetics, College of Pharmaceutical Sciences, Ritsumeikan University, Shiga, 525-8577, Japan

[3]Mitsubishi Tanabe Pharma Corporation, Yokohama, Aoba, Kamoshida, 227-0033, Japan

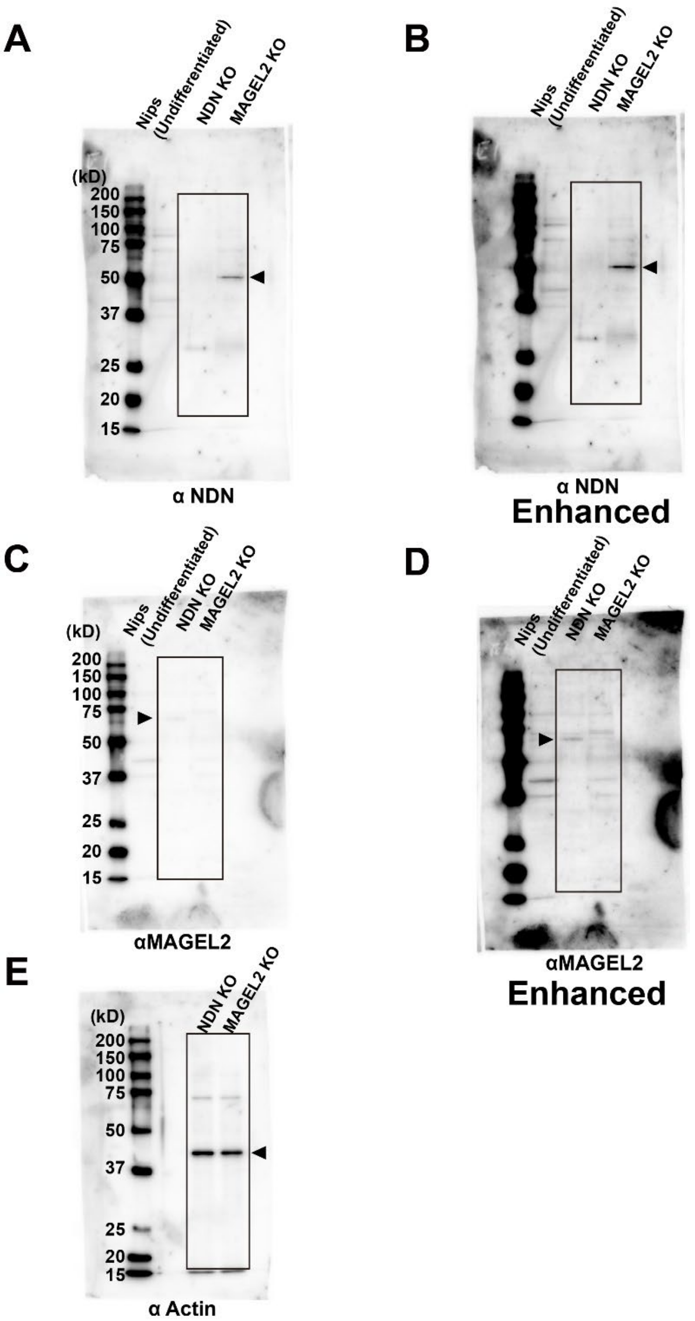

Figure S1. Original images of western blotting.

An original image of Fig.2C. For the final figures, Adobe Photoshop was used to crop the square area. The arrow heads indicated specific bands with the antibodies (NDN, MAGEL2, and Actin). (A)The band reacted with anti-NDN antibody in Nips (negative control) and NDN KO neuron, and MAGEL2 KO neuron extracts. (B)Enhanced blots of Fig. S1A. (C) The band reacted with anti-MAGEL2 antibody in Nips (negative control) and NDN KO neuron, and MAGEL2 KO neuron extracts. (D) Enhanced blots of Fig. S1C. (E) The band reacted with anti-Actin antibody in NDN KO neuron and MAGEL2 KO neuron extracts.

Abbreviation

NPTX1: Neuronal pentraxin 1

NLGN3: Neuroligin-3

NLGN4X: X-linked neuroligin 4

SLITRK1: Slit and Trk-like family member 1

SOX4: Sex determination region Y-box 4 protein

SEMA3B: Semaphorin 3B

MAGEL2: MAGE family member L2

MAP2: Microtubule-associated protein 2

SYN1: Synapsin 1

PSD95: Postsynaptic density protein 95

SCN: Sodium channel
